# "The Great Masquerader": An Interesting Case Series of Pulmonary Thromboembolism

**DOI:** 10.7759/cureus.32330

**Published:** 2022-12-08

**Authors:** Sri Balaji Sritharan, C. Poornima Raj, Vidya TA, Narendhiran Pandurangan, Vikrant Shinde

**Affiliations:** 1 Department of Internal Medicine, SRM Medical College Hospital and Research Centre, Chengalpattu, IND; 2 Department of Cardiology, SRM Medical College Hospital and Research Centre, Chengalpattu, IND

**Keywords:** pulmonary embolism (pe), venous thromboembolism (vte), case report series, acute management, high fatality, high clinical suspicion, varied clinical presentation

## Abstract

Venous thromboembolism (VTE) encompasses pulmonary embolism (PE) and deep vein thrombosis (DVT). The clinical manifestations of pulmonary embolism are highly variable and non-specific. We report five cases of pulmonary embolism, each with a unique clinical profile and degree of severity. The clinical, electrocardiographic, and radiologic findings of each patient are described in this case series along with the appropriate therapy based on hemodynamic stability. It is crucial to distinguish between hemodynamically stable and unstable pulmonary embolism and treatment should be started right away to reduce morbidity and mortality secondary to obstructive shock.

## Introduction

The “big three” cardiovascular disorders comprise venous thromboembolism (VTE), myocardial infarction and stroke. The incidence of PE ranges from 39 to 115 per 100,000 population annually [1]. Right ventricular failure due to acute pressure overload is the primary cause of death in severe PE. Chronic thromboembolic disease, chronic thromboembolic pulmonary hypertension (CTEPH), recurrent VTE and post-thrombotic syndrome (chronic venous insufficiency of the legs) are the main long-term consequences of VTE.

Pulmonary embolism is notorious for masquerading as other illnesses such as acute coronary syndrome, asthma, pneumonia, pleurisy, and congestive heart failure and often occurs concomitantly with asthma, heart failure and pneumonia, thereby confounding the diagnostic workup. The most useful approach is a clinical assessment of likelihood, based on presenting symptoms and signs, in conjunction with judicious use of laboratory testing and diagnostic imaging [2]. We present five such cases with varied clinical presentations.

## Case presentation

Case 1

**Figure 1:**
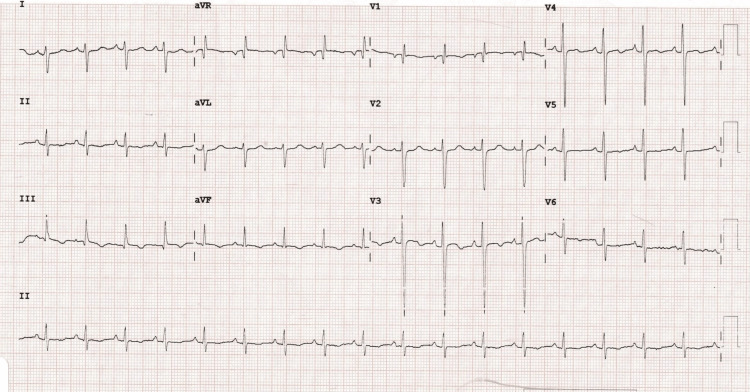
Standard 12-lead ECG showing sinus tachycardia, incomplete right bundle branch block, "S1Q3T3 pattern".

**Figure 2:**
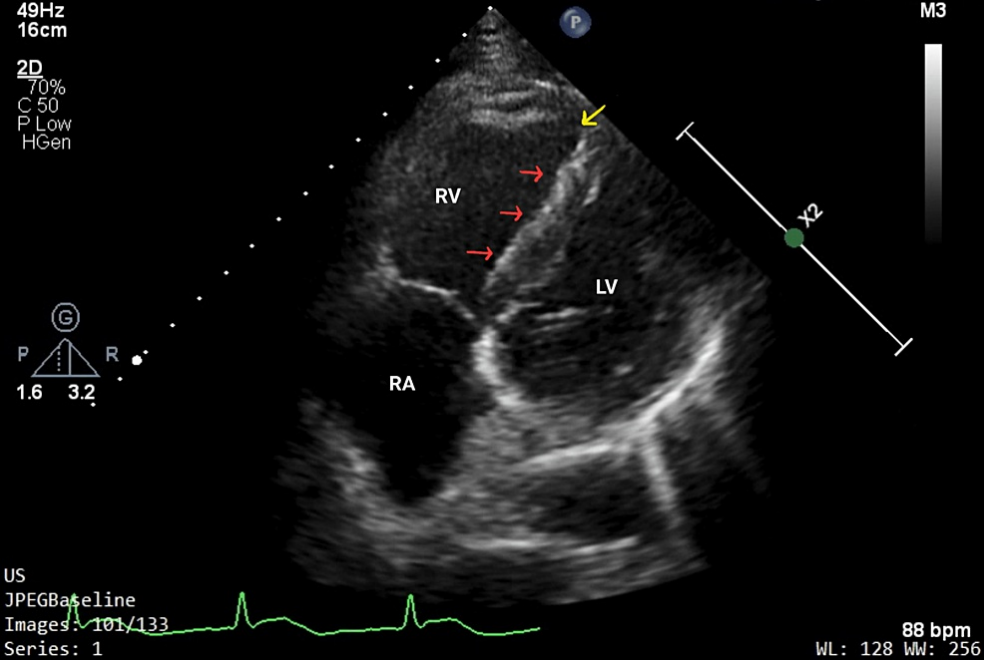
2D echo showing a dilated right atrium and right ventricle with a displaced interventricular septum (red arrows), "D-shaped left ventricle" and McConnell’s sign (yellow arrow).

**Figure 3:**
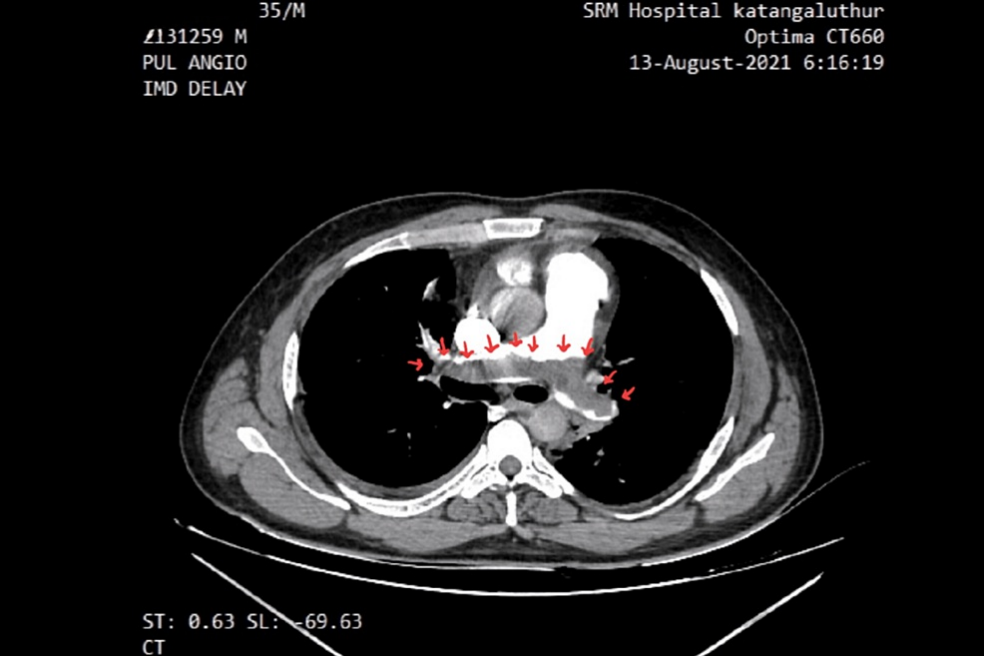
CT pulmonary angiogram showing a large intraluminal filling defect in the main pulmonary artery extending into the right and left pulmonary arteries causing near complete occlusion of the lumen (red arrows)

A 38-year-old obese male, a smoker with no known co-morbidities, presented with complaints of sudden-onset chest pain with breathlessness for four hours, associated with palpitations. The patient was tachypneic with blood pressure of 80/50 mm of hg with normal oxygen saturation. His ECG showed sinus tachycardia with the "S1Q3T3" pattern with right axis deviation and incomplete right bundle branch block (RBBB) (Figure 1). Troponin-I was elevated. The 2D echo revealed an underfilled left ventricle with a dilated right atrium and right ventricle (Figure 2). McConnell's sign-hyokinesia of free wall of RV with sparing of apex-was positive (Video 1). Computed tomography pulmonary angiography (CTPA) revealed a massive saddle thrombus in the bifurcation of the main pulmonary artery (Figure 3). This is a case of massive PE and the patient was thrombolysed with alteplase. Post thrombolysis, anticoagulation was started with heparin and later discharged with novel oral anticoagulant (NOAC) after clinical recovery. During follow-up, evaluation for thrombophilic states was done which revealed intermediate hyperhomocysteinemia (68 micromol/l) with normal vitamin B12, folate, protein C, and S levels as well as negative antinuclear antibody (ANA) and antiphospholipid antibodies (APLA) profile.

**Video 1:** McConnell's sign, i.e. hypokinesia of right ventricular free wall with sparing of the apex (yellow arrow).

Case 2

**Figure 4:**
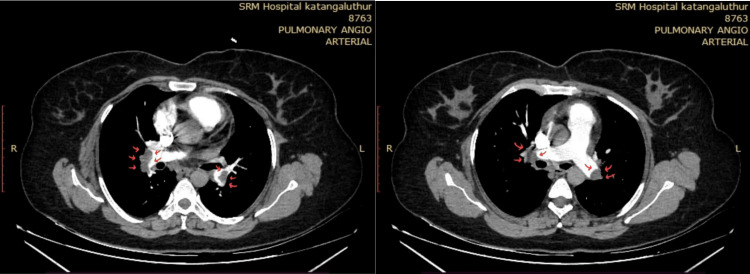
CT pulmonary angiogram showing partially occluding hypodense filling defects in right and left pulmonary artery and in segmental divisions (red arrows).

**Figure 5:**
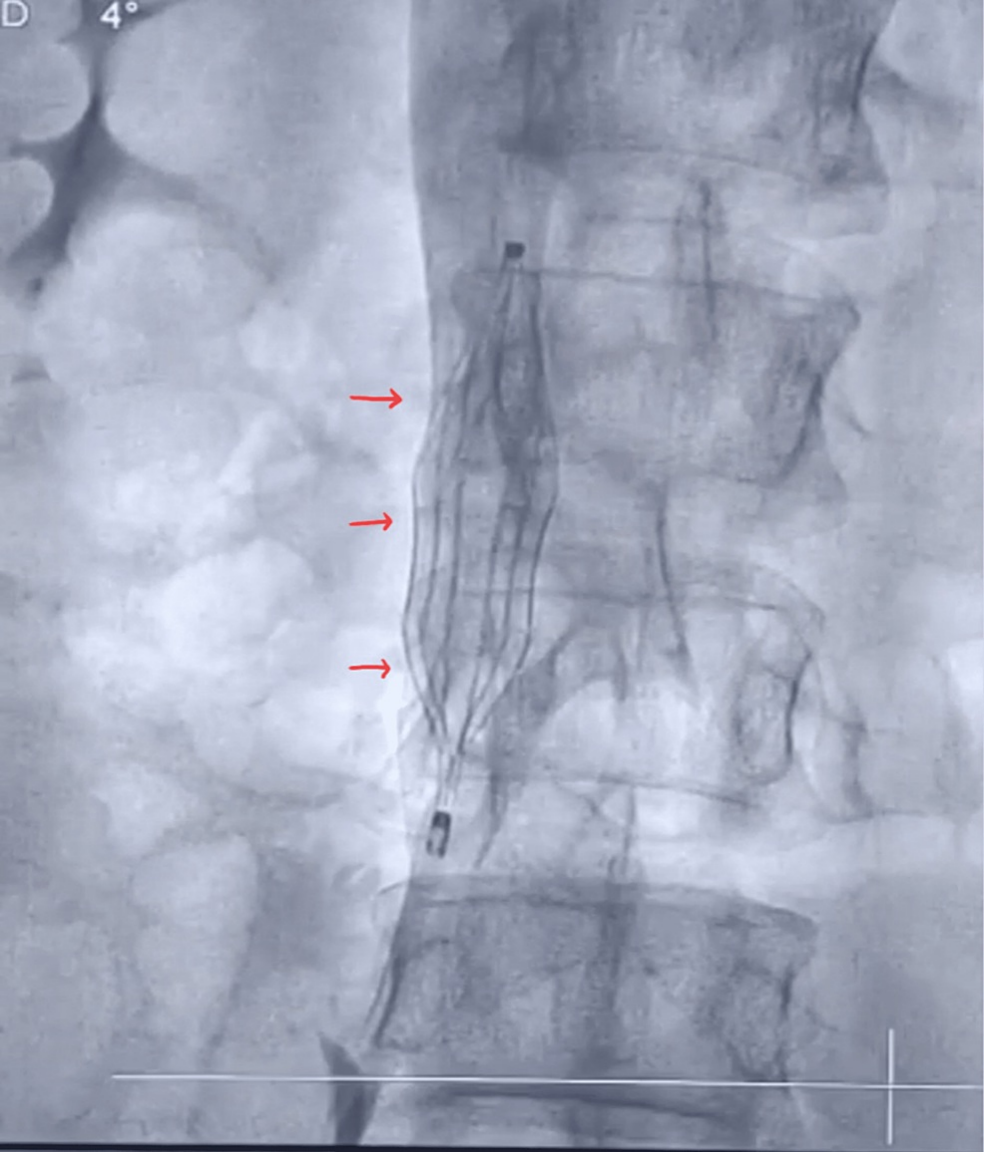
Inferior vena cava filter in the infra-renal position (red arrows). This reduces the chance of filtered thrombus occluding the renal vein.

A 45-year-old female with no known co-morbidities was admitted to the medical ward with complaints of left lower limb pain and swelling for one week. Venous Doppler of left lower limb revealed clots at calf and lower thigh. Initially, the patient was started with parenteral anticoagulation with low molecular weight heparin and warfarin was added on Day 3 and heparin was tapered off on Day 6 after achieving the therapeutic range of INR. Six days following admission, the patient developed complaints of breathlessness and palpitations. The ECG showed sinus tachycardia with 2D echocardiography revealing normal cardiac chambers with adequate ejection fraction. The CTPA shows filling defects in major pulmonary vasculature (Figure 4). Since the patient developed pulmonary thromboembolism (PTE) despite adequate anti-coagulation, an inferior vena cava (IVC) filter was placed for the patient (Figure 5). Procoagulant workup revealed normal protein C, S, antithrombin, and homocysteine levels with negative ANA and APLA profiles. Further evaluation showed the presence of heterozygous factor V Leiden mutation in the patient. On revisiting family history, her father revealed that he had DVT at 63 years of age (11 years previously) which was not further evaluated.

Case 3

**Figure 6:**
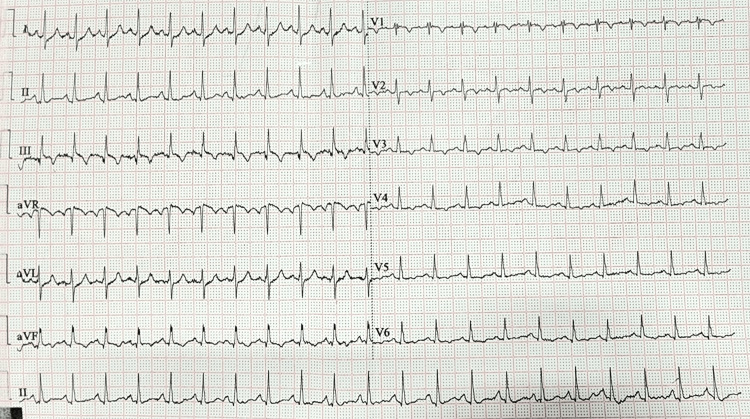
Standard 12-lead ECG showing sinus tachycardia of 142 beats per minute, S1Q3T3 pattern, right ventricular strain with T-wave inversion in V1-V4.

**Figure 7:**
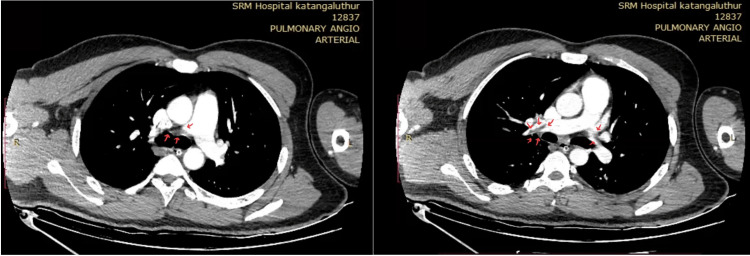
CTPA showing hypodense filling defects noted in the distal part of right and left pulmonary artery extending into the segmental branches.

A 28-year-old female presented to ER with complaints of giddiness followed by a fall and loss of consciousness (three episodes in a day), complaints of chest pain, palpitations, and breathlessness (NYHA class 2) for one day. She had a history of left lower limb swelling and pain in the calf region for one week. The patient has a known history of polycystic ovarian disease and was on treatment with cyproterone acetate2mg + ethinylestradiol (EE2) 35 mcg once daily for a month. On presentation, the patient had hypotension and hypoxemia and was put on supplemental oxygen therapy and inotropic support. ECG showed sinus tachycardia with an "S1Q3T3" pattern (Figure 6) and 2D echo showed dilated right atrium and ventricle with a normal left ventricle and ejection fraction. N-terminal-pro hormone brain natriuretic peptide (NT-proBNP) was grossly elevated. Lower-limb Doppler revealed left DVT and the CTPA showed acute PTE involving the right pulmonary artery and extension to segmental branches (Figure 7). This constituted a case of high-risk PTE. The oral contraceptive pill was stopped and the patient was given thrombolytic therapy with intravenous streptokinase 250,000 unit bolus followed by 100,000 units per hour continuous IV infusion for 12 hours. Post thrombolysis, the patient was started on anticoagulation with low molecular weight heparin. The patient was on regular follow-up with NOAC (apixaban) and anticoagulation was stopped after three months. 

Case 4

**Figure 8:**
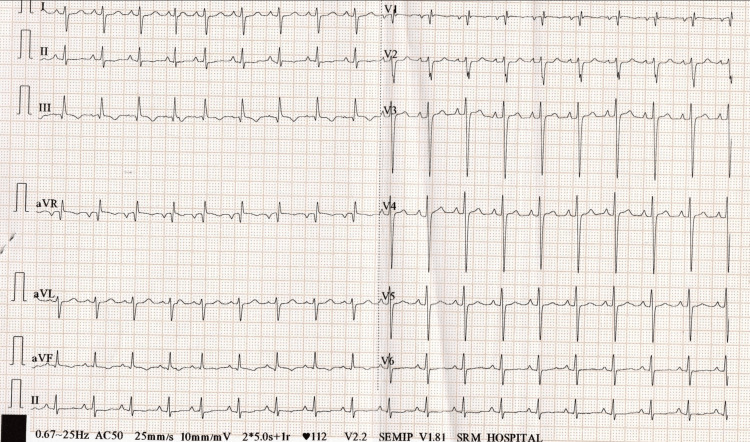
ECG showing sinus tachycardia and S1Q3T3 pattern

A 24-year-old male presented to ER with sudden onset breathlessness of class 2 NYHA for three days, palpitations and left lower limb claudication pain for five days. On initial examination, the patient had tachycardia, bilateral visible supra-clavicular pulsations, severe pallor, and systolic murmur in the pulmonary area radiating all over the precordium. The patient was hemodynamically stable. The ECG showed sinus tachycardia and an "S1Q3T3" pattern (Figure 8). The 2D echo showed dilated RA and RV with severe TR, severe pulmonary hypertension and adequate LV ejection fraction. Blood investigations showed severe anaemia with haemoglobin of 3.6g/dl. The D-dimer level was grossly elevated. Both arterial and venous doppler was done, which were normal. Evaluation for anaemia revealed microcytic hypochromic RBCs in peripheral smear with low serum iron, ferritin stores and high total iron binding capacity. The patient was a bodybuilder and was following severe diet restrictions. The CTPA showed an enlarged main pulmonary trunk with filling defects in the distal part of the right pulmonary artery and segmental and sub-segmental branches. This is a case of intermediate low-risk PE. The patient was given anticoagulation with heparin. Procoagulant workup during follow-up revealed a decrease in protein C and S levels with normal homocysteine and antithrombin levels as well as negative ANA and APLA profiles.

Case 5

**Figure 9:**
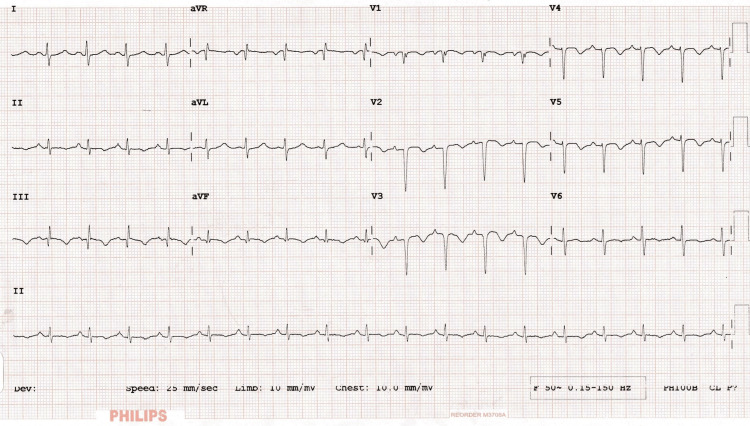
ECG showing sinus tachycardia, "S1Q3T3 pattern", and T-wave inversion in chest leads (V1-V6) and inferior leads.

**Figure 10:**
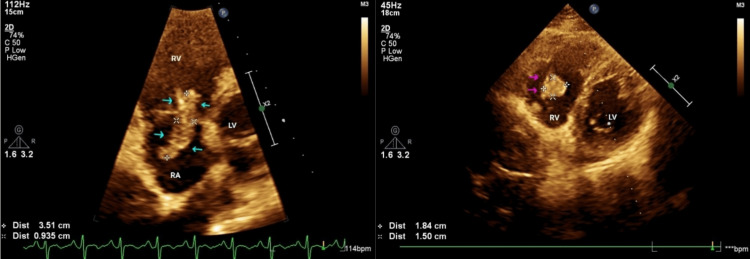
2D echo showing thrombus across tricuspid valve (cyan arrows) and RV thrombus (pink arrows).

**Figure 11:**
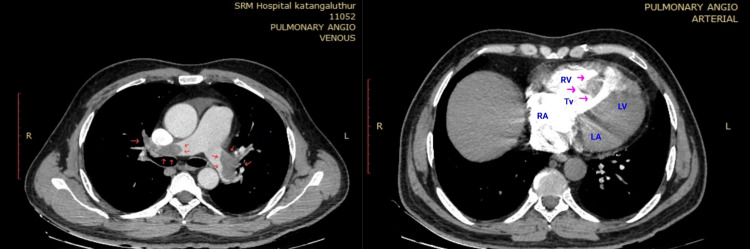
Irregular filling defects in right and left pulmonary artery (red arrows), intraluminal filling defect extending to the right ventricle (pink arrows).

A 42-year-old male with no co-morbidities "walked-in" to our outpatient department with breathlessness for five days and palpitations for one day. He had no other significant history nor predisposing risk factors. The patient was haemodynamically stable with tachypnea and tachycardia. The ECG showed sinus tachycardia with an "S1Q3T3 pattern" and T-wave inversion in chest leads and inferior leads (Figure 9). The patient was subjected to a 2D echo, resulting in surprising findings, showing dilated RA, RV and main pulmonary artery, RV dysfunction, severe pulmonary hypertension, moderate tricuspid regurgitation, and D-shaped left ventricle. There was a mobile thrombus across the tricuspid valve extending to the right ventricle (Figure 10) (Video 2, 3). Anticoagulation was started immediately with low molecular weight heparin (LMWH). Troponin I was elevated. The CTPA showed PE in pulmonary vasculature with a thrombus/clot in the right ventricle of the heart (Figure 11). Thrombolysis was started with streptokinase and the patient was planned for emergency mechanical thrombectomy. Unfortunately, the patient collapsed suddenly with hypotension and desaturation. Despite adequate resuscitation and supportive care, the patient could not be revived and was declared dead.

**Video 2:** Mobile thrombus across tricuspid valve extending to the right ventricle.

**Video 3:** Mobile thrombus in right ventricle resembling a characteristic "temple bell" movement.

## Discussion

VTE with PE is the third-most common acute cardiovascular syndrome worldwide after myocardial infarction (MI) and stroke [3]. VTE is considered to be an outcome of the interaction between setting-related temporary risk factors and patient-related permanent risk factors. The most common genetic causes of thrombophilia are factor V Leiden mutation and prothrombin gene mutation. Anti-phospholipid syndrome is the most common acquired thrombophilia. The other common risk factors are recent surgery, active cancer, prolonged immobilization, previous VTE, pregnancy, oral contraceptives, trauma, obesity, and heavy cigarette smoking. 

Except for Cases 3 and 5, where the patient's use of OCP was a predisposing risk factor, and where the patient experienced sudden death respectively, evaluation for hypercoagulable states was done in all other cases. During this evaluation, the patients were on NOAC (apixaban), as vitamin-K antagonists can alter Protein C and S levels. The cases presented here had varying causes such as hyperhomocystinemia, factor V Leiden mutation with DVT, OCP use and protein C and S deficiency.

Pathophysiology typically involves Virchow’s triad (stasis, vascular endothelial injury, hypercoagulable states) along with Infection and associated inflammation as key precipitants. The high recurrence rate of VTE in absence of anticoagulation supports the hypothesis that venous thrombosis can persist as a sub-clinical and perhaps chronic inflammatory state.

Symptoms and signs of PE are non-specific. Tachypnea is the most frequent sign, while dyspnea is the most typical symptom. Other symptoms include chest pain (pleuritic), anxiety, cough, haemoptysis, leg swelling, pain and rarely syncope. Out of the five cases reported here, the patient in Case 1 had symptoms similar to acute coronary syndrome, while the patient in Case 3 had a rare clinical presentation with syncope and other cases presented with dyspnoea with palpitations. Leg pain with/without swelling was present in three out of five patients. Case 5 had mild symptoms disproportionate to the severity of PE.

**Figure 12:**
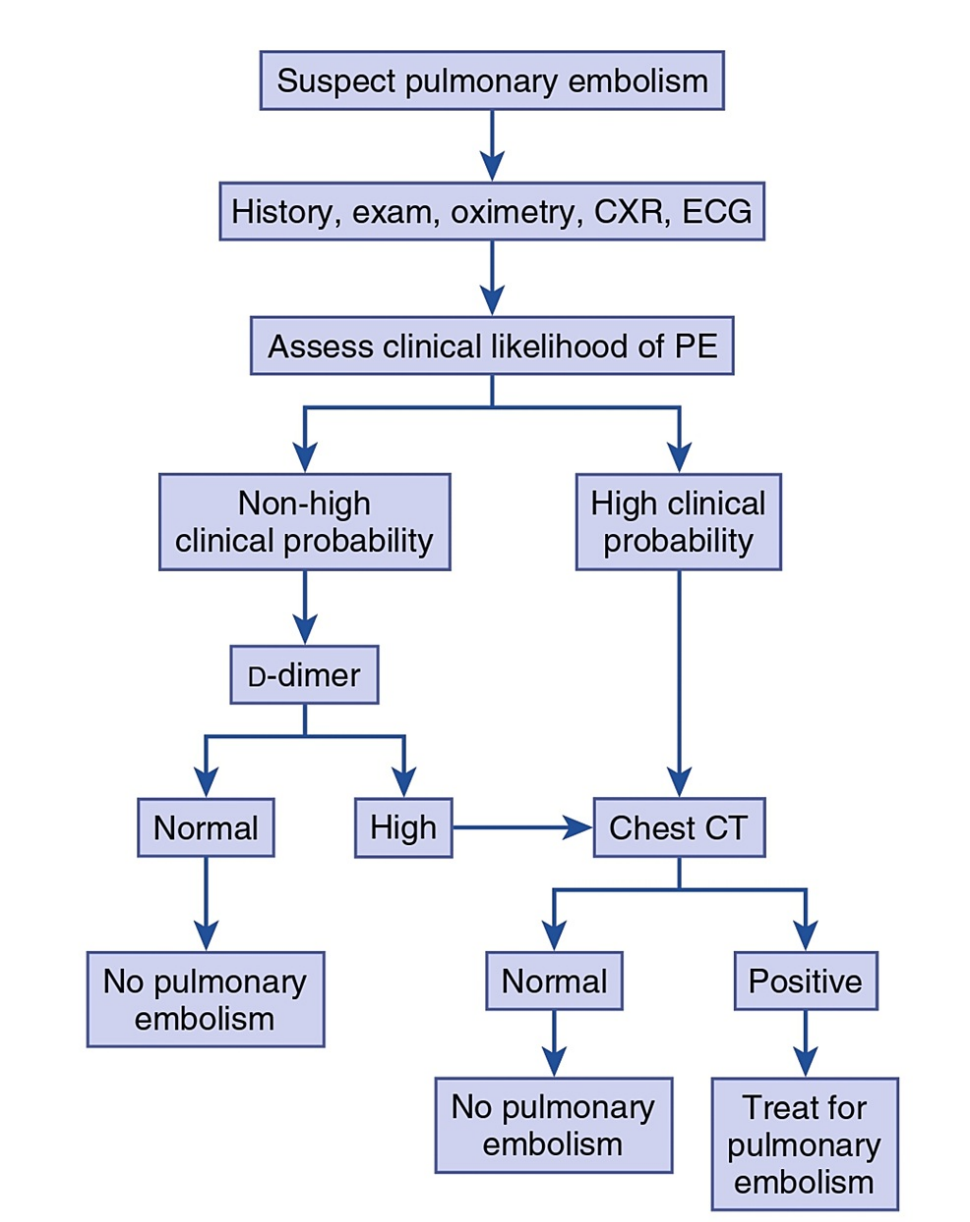
Integrated diagnostic approach for acute PE

Once pulmonary embolism is suspected, based on clinical presentation likelihood of PE is assessed by Well’s criteria as described in Table 1. For cases with a high or non-high clinical probability of suspected PE, laboratory testing and diagnostic imaging will be carried out accordingly (Figure 12).

**Table 1:** Well’s criteria for clinical assessment of PE; >4 points = high probability, ≤4 points = non-high clinical probability DVT: deep vein thrombosis, PE: pulmonary embolism

CRITERION	SCORING
DVT symptoms or signs	3
Alternative diagnosis less likely than PE	3
Heart rate > 100 beats/min	1.5
Immobilization or surgery within 4 weeks	1.5
Previous DVT or PE	1.5
Haemoptysis	1
Cancer treated within 6 months or metastasis	1

The D-dimer assay is a blood screening test with a strong negative predictive value in people with low clinical suspicion of PE. A negative D-dimer value rules out PE but the converse is not true. In absence of PE, D-dimer levels are elevated in the post-operative state, MI, major trauma, cancer, sepsis, and systemic illness. Although newer generation tests like latex agglutination or rapid ELISA with ‘sensitive D-dimer’ testing are preferred, adjusted D-dimer based on certain criteria like age-adjusted, YEARS algorithm, and clinical probability have been proposed for patients with low and intermediate probability of PE.

In PE, secondary to vascular obstruction pulmonary vascular resistance increases and causes pressure overload to the right ventricle. This releases cardiac bio-markers such as NT-proBNP, BNP and troponins. Three of our cases demonstrated elevated cardiac markers, two with troponin I and one with NT-proBNP. Elevated cardiac biomarkers increase the likelihood of adverse clinical outcomes.

The ECG in all five cases showed sinus tachycardia and four of them showed an "S1Q3T3" pattern. This pattern is the pathognomic sign of right heart strain which consists of a deep S in lead 1, a Q-wave and an inverted T wave in lead 3. Sometimes inverted T-wave in V1 to V4 and incomplete RBBB are also seen. Right heart strain can also be seen in cases of pulmonary hypertension, asthma and COPD in addition to PE.

Echocardiography is a rapid and practical tool for the detection of right ventricular overload, given by dilated RA, RV and dyskinesia or akinesia of the RV wall. Four of the five cases had characteristic Echo findings and the “McConnell sign” was seen in Case 1, which is characterised by regional wall motion abnormalities of the right ventricle with relative sparing of apex and base. Case 5 had a freely mobile thrombus in the RV across the tricuspid valve. 2D echo can also rule out close differentials such as MI or pericardial disease.

For patients with suspected PE, the CT scan serves as a prognostic and diagnostic test. Computed tomography pulmonary angiography is the investigation of choice for PE. With newer generation scanners, 3D images can be reconstructed and colour can be added to electronically enhance the details of thrombus localisation. CT can also pick up signs of right ventricular dysfunction. Occasionally alternate pulmonary abnormalities can be detected that may explain the symptoms and signs.

We had three high-risk and two intermediate low-risk cases out of these five cases. Stratification of acute PE based on haemodynamic stability and RV dysfunction is critical for prognostication and appropriate management (Table 2). Low-risk PE makes up for the majority of cases around 65% to 70% while 5% to 10% are high-risk. High-risk PE frequently manifests bilaterally, occasionally as a characteristic “saddle” thrombus in the bifurcation of the main pulmonary artery. Obstruction of more than one-third of the pulmonary vasculature is seen in intermediate-risk PE. The patient in Case 5 presented with normal hemodynamic status but was deemed to be "high risk" due to abnormal RV and elevated troponin I.

**Table 2:** Classification of acute pulmonary embolism

CARDIOLOGY (ESC, 2019)	AMERICAN HEART ASSOCIATION (AHA, 2011)	HEMODYNAMIC STATUS	PE SEVERITY INDEX (PESI) (OR SIMPLIFIED PESI)	EVIDENCE OF DYSFUNCTION	TREATMENT
High risk	Massive	Unstable	High	Typically abnormal right ventricle RV on imaging elevated troponin, OR both	Anticoagulation and advanced therapy
Intermediate-high risk	Submassive	Stable	High	Abnormal RV on imaging, AND elevated troponin	Anticoagulation with advanced therapy if clinical deterioration
Intermediate-low risk			High	May have abnormal RV on imaging OR elevated troponin BUT not both	Anticoagulation
Low risk	Low risk	Stable	Low	None	Anticoagulation with home therapy in a subset with reliable follow-up

Anticoagulation is the keystone of management in acute PE. In low-risk and intermediate low-risk cases, only anticoagulation is needed for treatment. In high-probability PE cases, anticoagulation should be administered immediately without any delay. The commonly used agents are unfractionated heparin (UFH), low molecular weight heparin (LMWH) and warfarin. Initiation of warfarin as monotherapy may paradoxically exacerbate hypercoagulability by decreasing endogenous anticoagulants protein C and S. This can be prevented by “bridging” warfarin with parenteral anticoagulants for at least five days after its initiation.

NOACs have a rapid onset of action, are prescribed in fixed dosages without the need for laboratory coagulation monitoring and have minimum drug-to-drug and drug-to-food interaction. NOACs are superior to warfarin for safety and non-inferior to warfarin for efficacy [4]. In a meta-analysis of 24,455 patients with acute VTE, NOACs compared with warfarin had a 40% reduction in major bleeding, 61% reduction in non-fatal intracranial bleeding and 64% reduction in fatal bleeding [5]. Three factor Xa inhibitors: apixaban, rivaroxaban, edoxaban and an oral thrombin inhibitor, dabigatran, are the approved NOACs for VTE management.

High-risk PE and intermediate high-risk PE require thrombolysis therapy in addition to anticoagulation. Fibrinolysis can be administered to patients up to 14 days following the onset of new symptoms. Intracranial haemorrhage is the most dangerous complication. The agents used are 100mg alteplase continuous infusion over 2 hours through the peripheral vein or intravenous streptokinase 250,000 unit bolus followed by 100,000 units per hour for 12-24 hours.

Other advanced therapies are catheter-directed fibrinolysis, pharmacomechanical therapy and mechanical embolectomy. For patients whose PE is resistant to thrombolysis or those who require the closure of the patent foramen ovale or surgical excision of the right atrial thrombus, surgical embolectomy can be an option as a rescue therapy. A surgical embolectomy was planned as an emergency procedure for Case 5 but the patient collapsed before the procedure could be carried out.

IVC filter is recommended in acute VTE with contraindications to anti-coagulation, recurrent PE in spite of adequate coagulation and as primary prophylaxis to high-risk VTE. IVC filters reduce the short-term risk of subsequent PE, increase the risk for DVT and have no impact on all-cause or PE-related mortality. When anticoagulation is deemed no longer necessary IVC filters should be retrieved as soon as possible to prevent complications such as filter migration and strut embolization [6].

Out of the five cases, three high-risk cases were given thrombolysis, one with alteplase and the other two with streptokinase. One case, despite adequate anti-coagulation, was having recurrent showers of PE and hence warranted IVC filter placement which was removed after six weeks. Lastly, the intermediate low-risk case required only long-term anticoagulation.

In Case 3, the patient had VTE secondary to OCP use. She was taking cyproterone acetate, a newer generation progestin with anti-androgenic activity together with EE2. The risk of VTE varies for different generations of OCPs. Higher the generation, the greater the risk of VTE. Compared to non-users, the risk of VTE in users of levonorgestrel with EE2 was threefold higher and the risk in users with cyproterone acetate and ethinylestradiol was sixfold [7]. The risk of VTE is highest for the first three months of initiation and decreases three months after stopping OCPs [8]. The patient in Case 3 developed VTE after one month of cyproterone acetate and EE2 use. A similar presentation has been reported by Kromm et al. where a 23-year-old female developed a stroke after three weeks of cyproterone acetate and EE2 [9].

Case 5 was a high-risk case which required advanced surgical therapy in addition to thrombolysis. The patient experienced sudden death as a consequence of obstructive shock and acute RV failure due to the dislodgement of RV thrombus into the pulmonary vasculature. The patient walked in to the outpatient department and died eight hours after admission as a consequence of massive PE with RV thrombus.

Adjunctive therapies include supportive care with analgesia, intravenous fluids, and oxygen. In an adequately anticoagulated patient, early ambulation is encouraged. Elastic-graded compression stockings are recommended in select patients with DVT to prevent post-thrombotic syndrome.

In regards to the optimum duration of anticoagulation, the 2019 European Society of Cardiology PE guidelines recommends against using the terms “provoked” and “unprovoked” when describing VTE in predicting the risk of recurrence [10]. The incidence of recurrent VTE has no correlation with the severity of the first event. It is of paramount importance to prevent recurrent VTE after PE due to high case fatality rates [11]. For the prevention of secondary VTE, patients have been classified into different groups based on their predisposing risk factors. Therapeutic anticoagulation for a minimum of three months of is recommended for all VTE patients [12]. Discontinuation of anticoagulation after three months is only advised for patients whose index PE was a consequence of a major transient risk factor [13]. Extended or indefinite anticoagulation is recommended for all other patient groups. Anticoagulation with NOACs is preferred over the "traditional" LMWH-warfarin regimen owing to the superior safety profile unless contraindicated. Aspirin is advised in patients who are not able to tolerate long-term anticoagulants or have increased bleeding risk.

## Conclusions

Pulmonary embolism has a wide range of clinical presentations, ranging from no symptoms to shock or sudden death. The majority of the patients present with dyspnoea, tachypnea or chest pain. However, some patients, even with massive pulmonary embolisms, have non-specific mild symptoms or are asymptomatic. Right ventricular failure as a sequel of PE can lead to death within the first few hours of presentation. Treatment in the emergency room is of paramount importance as RV failure is potentially reversible by restoring adequate blood flow through the pulmonary bed. If untreated, mortality is as high as 30% whereas it drops to 8% in treated PE. As initial clinical impressions are a cornerstone of diagnosis, it is imperative to have a high level of suspicion so that potentially curable patients are not missed.

## References

[1] Wendelboe AM, Raskob GE (2016). Global burden of thrombosis: epidemiologic aspects. Circ Res.

[2] Goldhaber SZ, Piazza G (2022). Pulmonary embolism and deep vein thrombosis: diagnosis. Braunwald’s Heart Disease.

[3] Raskob GE, Angchaisuksiri P, Blanco AN (2014). Thrombosis: a major contributor to global disease burden. Arterioscler Thromb Vasc Biol.

[4] Beyer-Westendorf J, Ageno W (2015). Benefit-risk profile of non-vitamin K antagonist oral anticoagulants in the management of venous thromboembolism. Thromb Haemost.

[5] van der Hulle T, Kooiman J, den Exter PL, Dekkers OM, Klok FA, Huisman MV (2014). Effectiveness and safety of novel oral anticoagulants as compared with vitamin K antagonists in the treatment of acute symptomatic venous thromboembolism: a systematic review and meta-analysis. J Thromb Haemost.

[6] Piazza G (2020). Advanced management of intermediate- and high-risk pulmonary embolism: JACC focus seminar. J Am Coll Cardiol.

[7] Lidegaard Ø, Nielsen LH, Skovlund CW, Skjeldestad FE, Løkkegaard E (2011). Risk of venous thromboembolism from use of oral contraceptives containing different progestogens and oestrogen doses: Danish cohort study, 2001-9. BMJ.

[8] World Health Organization Collaborative Study of Cardiovascular Disease and Steroid Hormone Contraception (1995). Venous thromboembolic disease and combined oral contraceptives: results of international multicentre case-control study. World Health Organization Collaborative Study of Cardiovascular Disease and Steroid Hormone Contraception. Lancet.

[9] Kromm J, Jeerakathil T (2014). Cyproterone acetate-ethinyl estradiol use in a 23-year-old woman with stroke. CMAJ.

[10] Konstantinides SV, Meyer G, Becattini C (2020). 2019 ESC Guidelines for the diagnosis and management of acute pulmonary embolism developed in collaboration with the European Respiratory Society (ERS). Eur Heart J.

[11] Carrier M, Le Gal G, Wells PS, Rodger MA (2010). Systematic review: case-fatality rates of recurrent venous thromboembolism and major bleeding events among patients treated for venous thromboembolism. Ann Intern Med.

[12] Boutitie F, Pinede L, Schulman S (2011). Influence of preceding length of anticoagulant treatment and initial presentation of venous thromboembolism on risk of recurrence after stopping treatment: analysis of individual participants' data from seven trials. BMJ.

[13] Agnelli G, Prandoni P, Becattini C (2003). Extended oral anticoagulant therapy after a first episode of pulmonary embolism. Ann Intern Med.

